# Frameless Stereotaxis for Subthalamic Nucleus Deep Brain Stimulation: An Innovative Method for the Direct Visualization of Electrode Implantation by Intraoperative X-ray Control

**DOI:** 10.3390/brainsci8050090

**Published:** 2018-05-15

**Authors:** Paolo Mazzone, Alessandro Stefani, Fabio Viselli, Eugenio Scarnati

**Affiliations:** 1 Stereotactic and Functional Unit, CTO Hospital, ASL Roma 2, Via S. Nemesio 21, 00145 Rome, Italy; 2 UOSD Parkinson, Department of Systems Medicine, University of Roma “Tor Vergata”, 00133 Rome, Italy; stefani@uniroma2.it; 3 UO Neurology, St. John the Baptist Hospital, ACISMOM, 00148 Roma, Italy; visellifabio@gmail.com; 4 Department of Biotechnological and Applied Clinical Sciences, University of L’Aquila, 67100 L’Aquila, Italy; scarnati@univaq.it

**Keywords:** deep brain stimulation, frameless neurosurgery, intraoperative X-ray control, Nexframe, subthalamic nucleus

## Abstract

The recent introduction of frameless devices has enabled stereotactic neurosurgery to reach a level of accuracy that is comparable to traditional frame-based methodologies. Among frameless devices, the Nexframe appears to be very useful in implanting electrodes into the subthalamic nucleus or other structures for deep brain stimulation in Parkinson’s disease. However, frameless devices, including the Nexframe, limit the possibility of intraoperative visual control of the placement of electrodes in the brain. Utilizing intraoperative O-arm Computed tomography (CT) scan or high-field Magnetic Resonance Imaging (MRI) could overcome this limitation, but their high cost restricts their use. Thus, in this paper we propose an innovation in Nexframe surgical planning that allows the intraoperative use of a C-arm X-ray apparatus to establish: (1) the progression of the electrode guide tube and the electrode in the brain; (2) the accuracy of the electrode trajectory; and (3) the correct attainment of the target. The proposed frameless technique using the Nexframe has been developed and successfully applied in our practice. It was shown to be helpful in overcoming the major issues that are usually encountered when electrodes are placed in the brain with frameless neurosurgery and reduced the risk of having to re-operate on patients to reposition the electrodes.

## 1. Introduction

Traditional frame-based stereotaxic surgery has long been regarded as a standard and reliable procedure for the precise targeting of deep brain structures, in particular the subthalamic nucleus (STN), in patients suffering from Parkinson’s disease (PD) [1]. Recently, this approach has evolved towards procedures that do not require the use of frames [2,3,4]. However, the utilization of frameless procedures raises a number of issues linked both to the accuracy of the electrodes’ placement and also to the way that it limits the possibility of having a direct intraoperative visualization of the electrode guide tube and the electrode itself as it advances towards the planned target [3,4].

Incorrect electrode placement, especially in the z coordinate and when an inclined trajectory is requested, may be subsequently evaluated with the aid of postoperative fusion images using computed tomography (CT) and magnetic resonance imaging (MRI), or clinically, when unwanted or unsatisfactory effects appear at the time of programming the stimulating device. Of course, unwanted effects cannot be evaluated under general anesthesia, especially if the implantation of the pulse generator is performed in the same surgical session in which the electrodes are implanted. If the misplacement of electrodes is detected postoperatively, new surgery for their proper repositioning may be necessary, causing further stress to the patient and surgeon.

Innovative technological tools, such as intraoperative O-arm and high-field MRI [4,5,6,7,8,9,10,11], whether or not they are associated with traditional neurophysiological recordings, may help in overcoming these issues, but their high costs limit their use.

**Figure 1:**
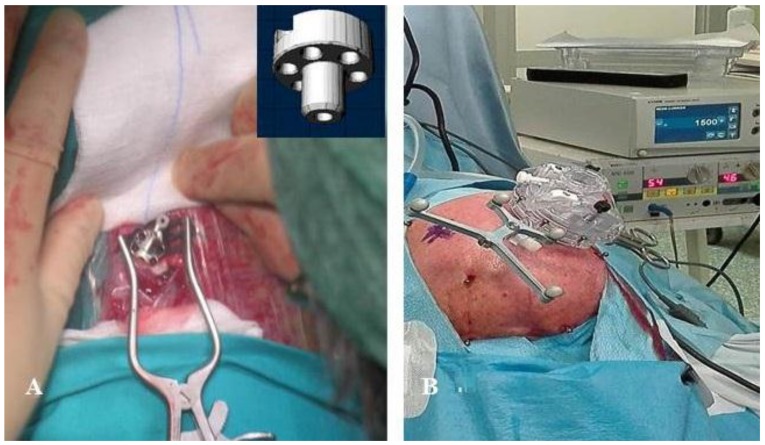
(**A**) The arch-less device, first used in 2001 [1], that could be applied to the skull using the Maranello Stereotactic System; (**B**) The Medtronic Nexframe DB2040.

With this in mind, we started our work with frameless functional neurosurgery in 2005 after ourselves developing a device that may be applied to the skull [12,13] (Figure 1A). Starting in 2016, we adopted the Nexframe^®^ (Nexframe^®^ DB2040; Medtronic Neurological Division, Minneapolis, MN, USA) for deep brain stimulation (DBS) of the STN (Figure 1B) [14,15,16]. This system is advantageous and safe for the implantation of electrodes in the STN, as reported by other groups [4,17,18,19]. The implantation of electrodes may be achieved without causing patients the discomfort produced by traditional frames that must usually be worn at least the night before surgery. The Nexframe may also be utilized if one wants to use intraoperative microrecordings of neuronal activity (IOMERs) [5] or somatosensory-evoked potentials (SEPs) for better identification of the structures that are to be targeted [20,21].

In this paper we describe an innovation in surgical planning that we developed using intraoperative X-rays in association with the Nexframe [4,22,23]. This innovation allows a faithful intraoperative visual verification of electrode placement and final position in the STN, and helps to overcome the major issues that arise when frameless surgery is performed.

## 2. Materials and Methods

### 2.1. Patients

Fourteen PD patients who underwent bilateral STN DBS using the Nexframe to treat rigidity and akinetic or dyskinetic symptoms were included in the study (Table 1). Of these, six patients (four males and two females), aged 65 ± 1.5 years (mean ± SD (Standard Deviation)), were implanted with 3389 quadripolar electrodes (Medtronic Neurological Division, Minneapolis, MN, USA) without intraoperative X-ray control. The remaining eight patients (five males and three females) aged 63 ± 3.4 years, were implanted with the aid of intraoperative X-ray control using a C-arm X-ray system. Of these, six patients were implanted with the quadripolar 3389 electrode and two with the octapolar electrode (Vercise lead DB 2201 ^TM^, Boston Scientific, Valencia, CA,USA). Surgery was performed under general anesthesia and was carried out using the Nexframe and the Stealth 7 neuronavigation system (Stealth 7^®^ Framelink Navigation Workstation, Medtronic, Minneapolis, MN, USA). SEPs were recorded intraoperatively through the electrode contacts as described in previous papers [20,21]. For all patients, a rechargeable pulse generator (Activa Rc, Medtronic Neurological Division, Minneapolis, MN, USA; or DB 1100 Vercise^TM^ , Boston Scientific, Valencia, CA,USA) was applied in a separate surgical session two to three weeks after electrode implantation.

### 2.2. Surgery Planning

**Figure 2:**
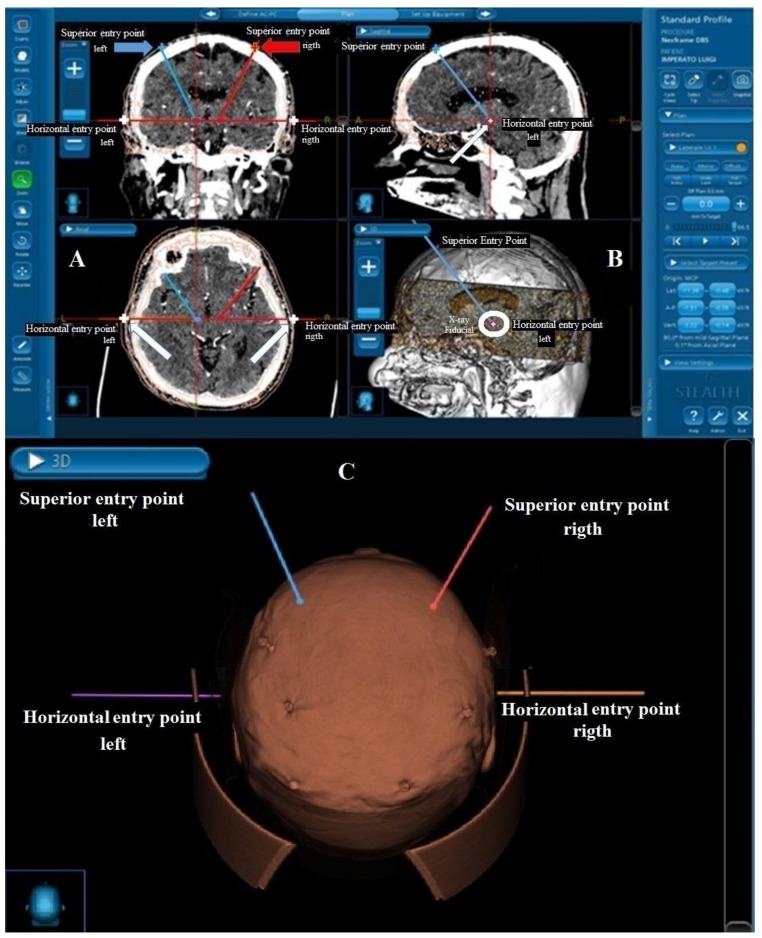
(**A**) Coronal (upper) and axial (lower) CT scan slides. The trajectories and superior entry points (red/blue arrows and red/blue cross) planned using the Medtronic Stealth-Station Framelink software package are reported. The inclined blue line represents the trajectory targeting the left STN, while the inclined red line represents the trajectory for the right STN. The horizontal red line crossing the target points from right to left is the Z plane. When this line crosses the skin (white arrows and cross) it is easy with the neuronavigational system to establish the horizontal entry point corresponding to the target point; (**B**) That which is reported in (**A**) is here represented in sagittal view. The projection of the horizontal entry point is represented by the red dot (white arrows and white cross inside the red dot). The inclined blue line represents the trajectory of the electrodes (upper slide); in (**B**), lower slide, a X-ray visible fiducial (white circle) is reported. (**C**) The 3D representation of the surgical planning and fiducial screws.

The Stealth computerized system for image fusion (Medtronic Stealth-Station Framelink^®^ Software package) was employed using angio-CT scan images taken the day before surgery and fiducial marker screws (Medtronic, Minneapolis, MN, USA) applied to the skull. Fusion images were obtained from 1 mm thick angio-CT scan slices and from T1- and T2- weighted MRI slides Traditional coordinates and trajectories were adopted to target the STN, i.e., 12 mm lateral with respect to the midline for the X coordinate; −2 mm with respect to the midcommissural point (also taking into account the individual anatomo-radiological variability) for the Y coordinate, and 4 mm below the Ca–Cp line for the Z coordinate. The planned trajectories were always extraventricular and the superior entry point of the electrodes was checked in the surgical space using the facility that the Nexframe offers to verify the inclination of the electrode trajectory. Particular attention was paid to the angiographic representation of cortical vessels to ensure that there was no conflict between the electrode trajectory and vessels. Once the two STN targets and their representation on the axial CT/MRI fusion images had been located, a bilateral horizontal entry point that corresponded to the z coordinate of the two electrodes along a horizontal line was added to the established surgical plan (Figure 2).

### 2.3. Innovative Tools

Two innovative tools, conceived and developed by one of the authors (PM), were employed

**Figure 3:**
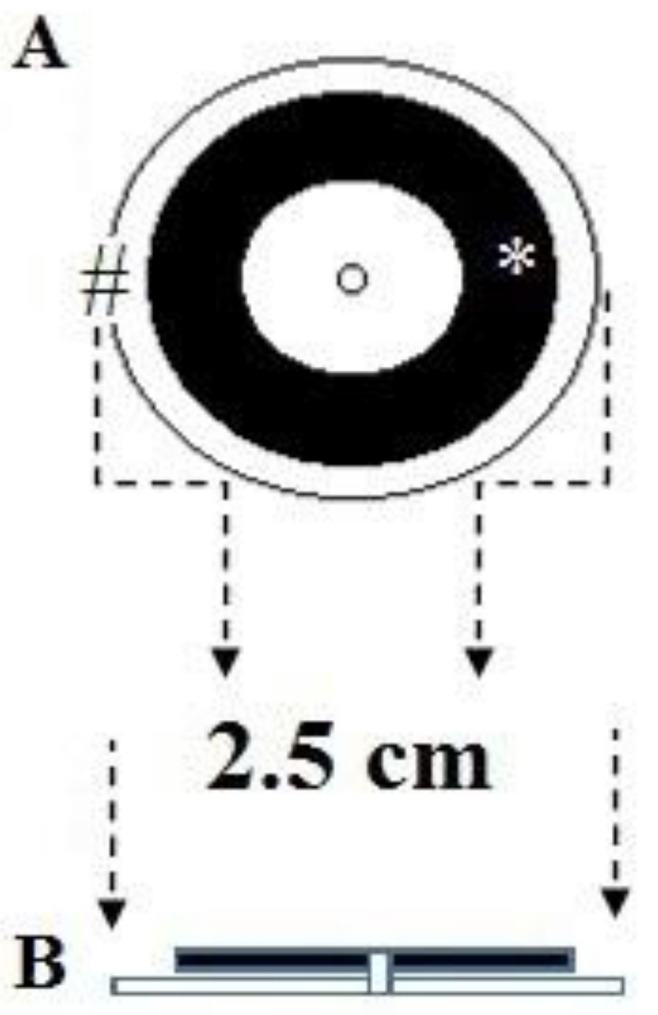
(**A**) Schematic drawing of the radiopaque fiducial flat ring and (**B**) the plastic support; in the center there is the hole for the navigator probe, (*) sagittal view; (#) axial view.

**Figure 4:**
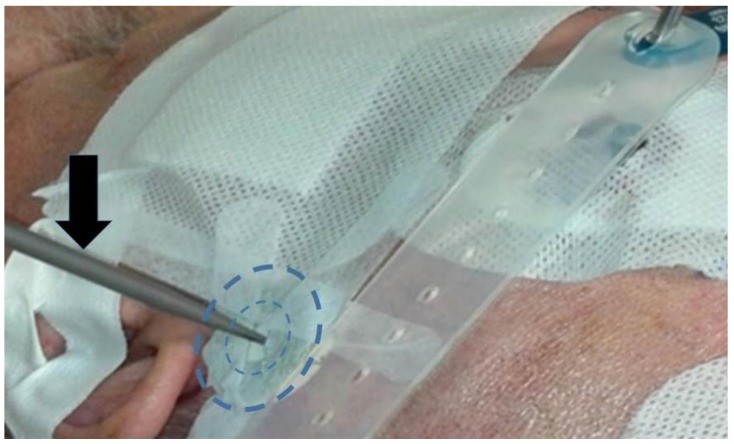
The atraumatic neuro-navigational application of radiopaque circular target fiducials. The navigator probe (in grey) indicates the horizontal entry point (black arrow). The dashed circles represent the profile of X-rays-visible fiducials (see also Figure 3).

**Figure 5:**
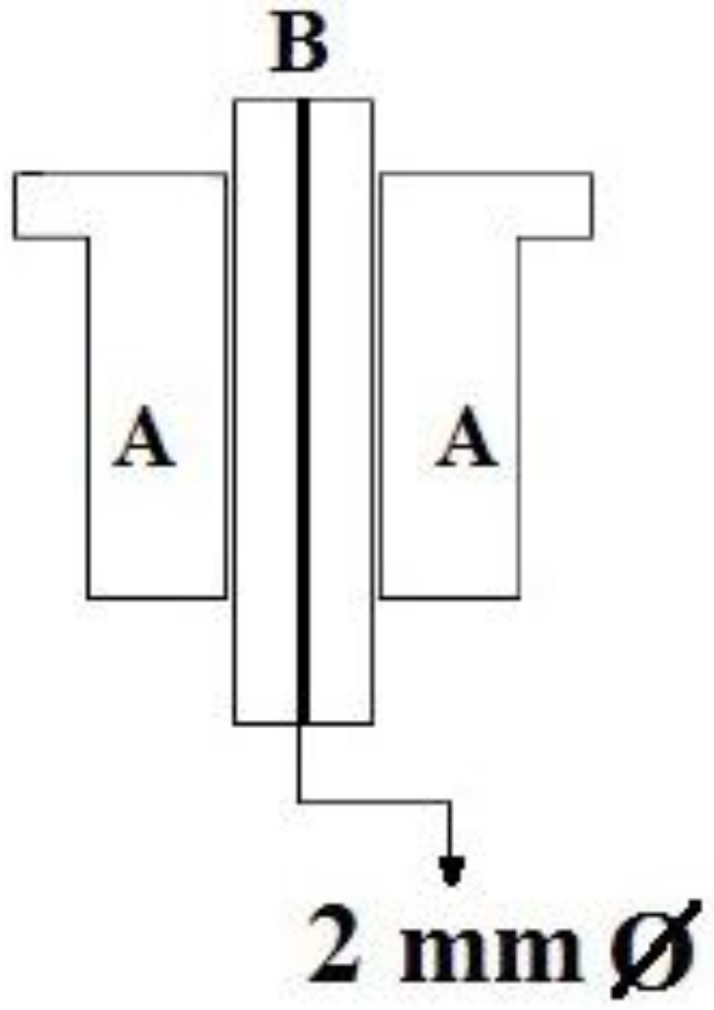
(**A**) Schematic drawing representing a sagittal section of the cylindrical rocket assembly made of amagnetic stainless steel. The central cylinder can be slided to change its distance from the skull surface and to adjust the distance between the 0 point and the target. (**B**) In black: the central track to drill the hole and to guide the electrode until the brain target (see also Figure 6).

**Figure 6:**
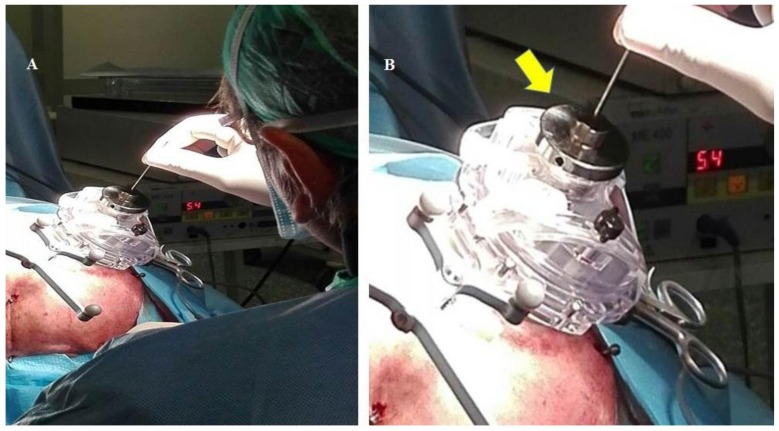
The Nexframe modified technique. (**A**) after establishing the trajectory inclination the stainless steel cylindrical rocket is inserted In the Nexframe tower. This allows a 2 mm Ø burr hole to be drilled, through which the electrode guide tube and the electrode may be inserted into the brain. (**B**) Detail of the stainless cylindrical rocket, (yellow arrow) during the insertion of the electrode guide tube (see also Figure 4).

(a) A system of two fiducials visible to X-rays, which were used to align the two STN target points under intraoperative X-ray control. Each fiducial was a flat ring of radiopaque metal with an external diameter of 2.5 cm (Figure 3 and Figure 4). It was mounted on a plastic ring support provided with a central hole of the same size as the probe employed to determine the horizontal entry point line (Figure 3 and Figure 4).
(b) An amagnetic stainless steel cylindrical rocket (Figure 5 and Figure 6). The rocket was designed to fit inside the tower body of the Nexframe. A second amagnetic stainless steel cylinder was fitted into the central part of the cylindrical rocket. Its height was variable with respect to the bone plane and the Nexframe tower body level. It served to measure the distance of the 0 plan from the target point. Inside the second cylinder a circular track with a diameter of 2 mm allowed a precise hole for inserting the electrode guide tube to be drilled with a calibrated bit (Figure 5 and Figure 6).

### 2.4. Surgical Procedure

The patient was secured to the bed in a supine position, the head was fixed using a Mayfield apparatus, and each fiducial screw was put in contact with a passive planar registration probe equipped with reflective spheres that could be tracked by the cameras of the Stealth Station^®^. The superior entry points of the electrodes were marked on the scalp and the head of the patient was prepared using sterile bands. Then, the horizontal entry points were checked using the same method and the X-ray-visible ring was bilaterally applied to the skull skin with adhesive Steri Drape (3M Milan, Italy) (Figure 4). The center of this ring represented the target point of the electrode and allowed the accuracy of the electrode placement to be verified intraoperatively using X-rays, just as in frame-based procedures (Figure 4). After the incision of the skin and positioning of the Nexframe platform, each fiducial marker screw for neuronavigation was put in contact with the registration probe through the drape to again perform registration, according to the literature [2,3,4]. Then, the Nexframe platform was adjusted to orient the trajectory according to the planned target, using the guidance provided by the FrameLink software. An amagnetic, custom-made, stainless steel drill bit was then used to drill a burr hole (Ø 2.0 mm) to allow the penetration of the electrode guide tube and electrode (Figure 6), as previously reported [21].

### 2.5. Data Evaluation

The differences between the real values of X, Y and Z coordinates with respect to the planned values were evaluated in the patients in which intraoperative X-ray control was performed and compared to those in which no intraoperative control was used (Table 1). This was done using the Stealth 7^®^ Framelink Workstation for image fusion and taking into account pre- and post-operative CT scans and MRI slides. Clinical evaluations, using the Unified Parkinson’s Disease Rating Scale (UPDRS) Part III, were pre- and postoperatively performed with DBS ON in the eight patients implanted with intraoperative X-ray control and in the six patients implanted without intraoperative X-ray control (Table 2). We also evaluated variations in the severity of L-dopa-induced dyskinesias (LIDs) according to UPDRS Part IV and the number of days required to establish the stimulation parameters giving the best clinical outcome. Statistical analysis was performed with a two way ANOVA and values are reported throughout as mean ± SD. The Statistica 8 software package was employed.

## 3. Results

**Figure 7:**
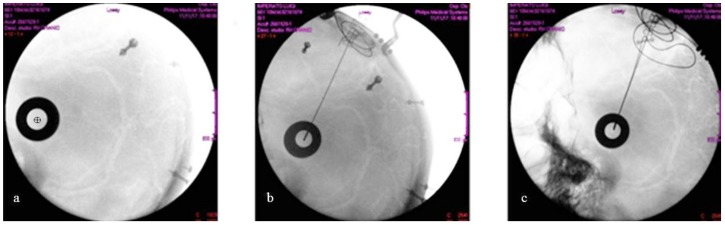
(**a**) The latero-lateral X-ray image shows the alignment of the radiopaque fiducials. The screws applied to the skull for navigation are also visible. The centre of the circle (black cross) represents the target (i.e. the horizontal entry point). (**b**) The right electrode hits the target. (**c**) The final intraoperative control with the two electrodes (latero-lateral X-ray image), after their fixation to the skull with titanium microplates.

**Figure 8:**
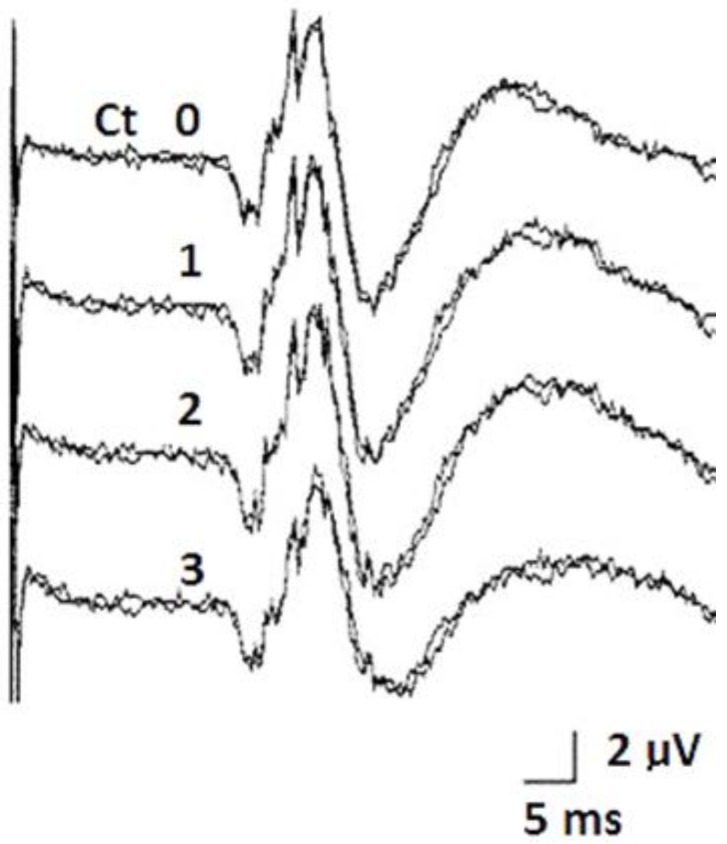
The somatosensory-evoked potentials that can be intraoperatively recorded from the quadripolar 3389 Medtronic electrode in the STN. Contact 0 is the deepest of the four contacts.

No asymmetry concerning the position of the distal tip of the two electrodes was detected intraoperatively (Figure 7) in the group of patients implanted under X-ray control, thus the electrodes were correctly positioned and symmetric when compared to each other. Their correct placement in the STN was further supported by the fact that the typical SEP waves that may be recorded in the STN through the electrode contacts (Figure 8) were found in each patient.

**Figure 9:**
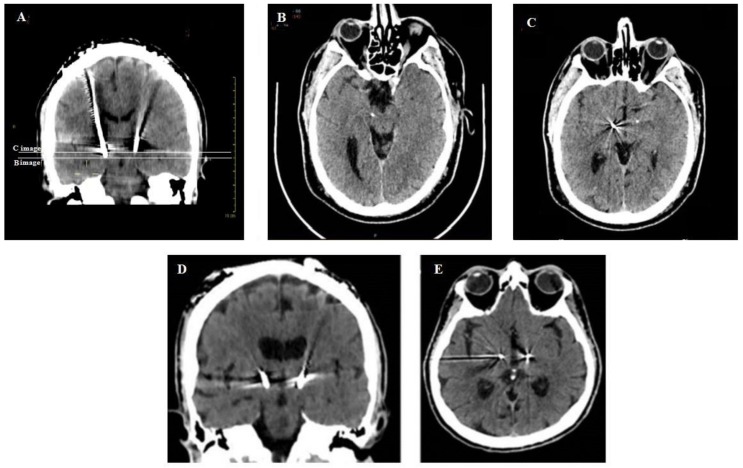
A representative comparison in postoperative CT scans of a patient implanted without intraoperative X-rays control (**A**–**C**) with a patient implanted with intraoperative X-rays control (**D**,**E**). The coronal CT-scan shows the different depth of electrodes in the STN; the white lines correspond to the axial levels of the CT scan reported in B and C. The distal tips of the electrode in the right and left STN are visible in B and C, respectively. The contact 1 of the electrode in the right STN and the distal tip of the electrode in the left STN are visible in C. The correct trajectory of the two electrode and the precise correspondence of their tips in a patient implanted under X-ray control are visible in the coronal (**D**) and axial (**E**) CT scans.

CT scans showing the depth of the electrodes in patients implanted without intraoperative X-ray control were compared with patients implanted with intraoperative X-ray control (Figure 9).

The differences between the planned and actual coordinates were measured and compared in the two groups of patients. The correspondence between the planned and actual coordinates was more precise in patients implanted with the X-ray control than in patients who were postoperatively controlled by CT scan or MRI. The main differences concerned the Y and Z coordinates, where the variations were significantly greater in patients implanted without intraoperative X-ray control (Table 1).

In all patients, DBS delivered under appropriate electrical parameters and electrode configuration produced an effective and reliable control of major parkinsonian symptoms. However, the search for optimal parameters for the best clinical outcome required a significant higher number of days in patients implanted without X-ray control than in patients implanted with X-ray control (Table 2).

## 4. Discussion

The implantation of electrodes for bilateral STN DBS using frameless stereotaxy has provided satisfactory results in PD patients showing rigidity and akinetic–dyskinetic symptoms [3,4,14,15,17]. For many years in our practice we implanted electrodes using the Maranello frame-based stereotactic system (CLS Titanium, Forlì, Italy) with patients under either local or general anesthesia, also recording IOMERs or SEPs [1,20,21]. Satisfactory clinical outcomes were obtained and the precision and accuracy of the electrode positioning fully satisfied our expectations [21]. Since frameless surgery is better tolerated by patients than frame-based surgery, we started to use the Nexframe three years ago. Important advantages of frameless neurosurgery are reduced discomfort for the patients, who tolerate the surgery better, since they are no longer required to wear the traditional frame that blocks the head for a long time, and for the neurosurgeon, since she/he may benefit from a more efficient workflow, leading to decreased time in the operating room. In frameless neurosurgery, the stereotactic frame is replaced by skull-bone-implanted fiducial markers and by a burr hole guidance device [2,3,4,12].

The clinical outcomes that may be obtained with electrodes implanted with the Nexframe are comparable to those obtained with traditional frame-based surgery. The Nexframe has been compared to the Leksell, the Cosman–Robert–Wells and the Fisher stereotaxic frames [14,15,16,18,19]. In these studies, no substantial difference in the improvement of the UPDRS-III score and reduction in LIDs was found between patients implanted with the Nexframe and those implanted with frame-based surgery [17].

When dealing with the Nexframe, we preferred general anesthesia and recording only SEPs for electrophysiological identification of the STN to reduce patient discomfort and the duration of surgery. After three years of using the Nexframe we have reached the same levels of accuracy and good clinical outcomes as with traditional frame-based methods for STN DBS.

Starting from the second half of 2017, we introduced X-ray control in our practice to verify the positioning of the electrode tube guide and the electrode in the brain intraoperatively. We did this because postoperative CT scans and MRIs of patients who had been implanted with the Nexframe elsewhere and later brought to our attention, as well as some of our own early patients, frequently showed a difference between planned and actual electrode position. This difference mainly concerned the Z coordinate (electrode depth) and, to a lesser extent, the X and Y coordinates.

According to the neurologists who examined these patients (FV and AS), these differences could have contributed to the difficult and time-consuming setting and resetting of stimulation parameters that was requested in these patients. The resolution of these issues in some patients required a new surgical session for the repositioning of the electrodes.

To overcome these issues, we introduced an innovation to the surgical technique that does not modify the method of using the Nexframe but offers immediate visual control of the accuracy of the electrode placement as soon as it is advanced in the brain. This innovation does not add any surgical risk or require additional time compared to traditional frameless surgery. In addition, we developed a tool to drill a hole in the skull with a diameter of exactly 2 mm, which greatly limits the risk of causing electrode movement and brain shift, guaranteeing greater precision in electrode positioning. Moreover, since our technique utilizes a well-calibrated drill hole and a straight track guided by the internal cylinder of the rocket assembly and the electrode tube guide, a substantial deviation of the electrode from the planned trajectory becomes virtually impossible. The possibility of using traditional X-ray-visible fiducials to provide intraoperative control of the trajectory and symmetry of the electrodes allows better correspondence between the planned and realized coordinates. In this way the accuracy of the electrode’s position may easily be verified when the electrode is placed, and not later using postoperative CT scans or MRIs [6,12,17,24]. This decreases the risk of additional surgery for electrode repositioning. In addition, possible changes to the electrode alignment during the surgical procedure that may be caused by the presence of a hemorrhagic bleeding can be promptly verified before awakening the patient, without waiting for postoperative CT scans and MRIs.

The surgical session to implant the electrodes for STN DBS with the Nexframe assisted by intraoperative X-ray control may be completed in three to four hours, thus no additional time is required compared to traditional frame-based procedures. In addition, the method that we adopted allows us to plan and perform surgery on the same day. The success of this technique has convinced us to renounce the use of traditional stereotaxic frames in the last two years.

No significant difference was found in the Z coordinate in our patients when comparing the intraoperative X-rays controls to postoperative CT scans. Most likely, this was also due to our electrode fixation method, which uses titanium microplates (see the picture in Figure 8c, which was taken soon after the final electrode was fixed to the skull). The differences concerning the X and Y coordinates may have been caused by differences in the Z coordinate (depth) and might have been enhanced given the trajectory inclination along the anteroposterior and lateral directions. We have not considered anteroposterior X-ray controls for two reasons: firstly, because the X and Y coordinates are strictly dependent on the predetermined choice of the entry point and the trajectory inclination, and secondly because the presence of the Mayfield apparatus and the Nexframe tower do not allow for the alignment of the anteroposterior plane to take X-ray slides.

## 5. Conclusions

In conclusion, the implantation of electrodes in the STN for DBS in PD simply using frameless stereotaxy, intraoperative X-ray control, general anesthesia and SEP recording [3,4,17,20,21,22] may reveal inaccuracies in the electrode positioning and risks of adverse events in real time and may produce consistent motor outcomes from six to twelve months after starting DBS [3,4,17] that are comparable to those obtained using more complex, frame-based DBS [1,4,12,21,22].

## Figures and Tables

**Table 1:** Differences in mm between the planned and actual X, Y and Z coordinates. The differences in the depth of the electrode tips in patients implanted without X-ray control, reported in the grey column, were measured in postoperative Computed Tomography (CT) scans. (* *p* < 0.001 comparing Y and Z values in the two groups of patients, two-way ANOVA).

Patients Implanted with X-ray Control	X	Y	Z	Patients Implanted without X-ray Control	Difference in the Depth of Right and Left Electrode	X	Y	Z
1	0.9	0.5	0.5	1	4	1.5	2.1	3.5
2	0.9	2.4	0.2	2	1	1.0	2.3	3.0
3	1.5	3.3	0.5	3	2	0.5	3.4	2.0
4	0.4	3.2	0.7	4	1	1.6	2.2	3.1
5	0.2	1.1	0.6	5	3	1.5	2.5	3.2
6	2.2	1.3	0.3	6	3	0.7	3.1	2.8
7	1.0	0.5	0.6	Mean ± S.D (mm)	2.3 ± 1.2	1.1 ± 0.5	2.6 ± 0.5	2.9 ± 0.5
8	0.5	1.3	0.5					
Mean ± SD (mm)	0.95 ± 0.76	1.87 ± 1.0 *	0.48 ± 0.1 *					

**Table 2:** Clinical evaluation. Patients were evaluated before surgery and after one year of STN Deep Brain Stimulation (DBS) (* *p* < 0.01 postoperative vs preoperative; ** *p* < 0.05 postoperative vs preoperative; *** *p* < 0.01 X-ray control vs no X-ray control; two-way ANOVA all comparisons).

	Preoperative UPDRS III DBS ON	Postoperative UPDRS III DBS ON	Preoperative LIDs DBS ON	Postoperative LIDs DBS ON	Preoperative L- Dopa mg/die	Postoperative L-Dopa mg/die	Days Required in Programming DBS Setup
Patients implanted with intraoperative X-ray control	25 ± 6.1	14 ± 4.5 *	3.3 ± 0.9	1.8 ± 0.8 **	1.150 ± 227	895 ± 165	4 ± 3 ***
Patients implanted without intraoperative X-ray control	26 ± 3.6	16 ± 3.9 *	3.6 ± 0.5	2.3 ± 0.5 **	1.240 ± 461	1.040 ± 270	10 ± 3
